# MambaKAN: An Interpretable Framework for Alzheimer’s Disease Diagnosis via Selective State Space Modeling of Dynamic Functional Connectivity

**DOI:** 10.3390/brainsci16040421

**Published:** 2026-04-17

**Authors:** Libin Gao, Zhongyi Hu

**Affiliations:** 1 Artificial Intelligence College, Zhejiang Industry & Trade Vocational College, Wenzhou 325000, China; gaolibin@zjitc.edu.cn; 2 College of Computer Science and Artificial Intelligence, Wenzhou University, Wenzhou 325035, China

**Keywords:** Alzheimer’s disease, dynamic functional connectivity, state space model, Mamba, Kolmogorov–Arnold network, variational autoencoder, interpretability, resting-state fMRI, deep learning, brain connectivity

## Abstract

**Background/Objectives:** Alzheimer’s disease (AD) is an irreversible neurodegenerative disorder that imposes a profound burden on global public health. While resting-state functional magnetic resonance imaging (rs-fMRI)-based dynamic functional connectivity (dFC) analysis has demonstrated promise in capturing time-varying brain network abnormalities, existing deep learning methods suffer from three fundamental limitations: (1) an inability to model temporal dependencies across dynamic connectivity windows, (2) reliance on post hoc black-box explainability tools, and (3) misalignment between feature learning and classification objectives. **Methods:** To address these challenges, we propose MambaKAN, an end-to-end interpretable framework integrating a Variational Autoencoder (VAE), a Selective State Space Model (Mamba), and a Kolmogorov–Arnold Network (KAN). The VAE encodes each dFC snapshot into a compact latent representation, preserving nonlinear connectivity patterns. The Mamba encoder captures long-range temporal dynamics across the sequence of latent representations via input-selective state transitions. The KAN classifier provides intrinsic interpretability through learnable B-spline activation functions, enabling direct visualization of how latent features influence diagnostic decisions without post-hoc approximation. The entire pipeline is trained end-to-end with a joint loss function that aligns feature learning with classification. **Results:** Evaluated on the Alzheimer’s Disease Neuroimaging Initiative (ADNI) dataset across five classification tasks (CN vs. AD, CN vs. EMCI, EMCI vs. LMCI, LMCI vs. AD, and four-class), MambaKAN achieves accuracies of 95.1%, 89.8%, 84.0%, 86.7%, and 70.5%, respectively, outperforming strong baselines including LSTM, Transformer, and MLP-based variants. **Conclusions:** Comprehensive ablation studies confirm the indispensable contribution of each module, and the three-layer interpretability analysis reveals key temporal patterns and brain regions associated with AD progression.

## 1. Introduction

Alzheimer’s disease (AD) is a progressive neurodegenerative disorder characterized by irreversible cognitive decline, memory impairment, and behavioral dysregulation [1]. Its neuropathological hallmarks—amyloid-β plaque accumulation, neurofibrillary tau tangles, synaptic loss, and cortical atrophy—manifest insidiously over years before clinical symptoms appear, making early detection essential yet difficult [2]. The global prevalence of dementia, predominantly driven by AD, has exceeded 55 million individuals and is projected to reach 152 million by 2050 [3], imposing substantial healthcare and socioeconomic burdens worldwide [4].

The AD continuum spans from cognitively normal (CN) individuals to early mild cognitive impairment (EMCI), late mild cognitive impairment (LMCI), and clinical AD. Approximately 10–15% of MCI patients convert to AD annually, and early intervention at the MCI stage can meaningfully slow disease progression [5]. However, the subtle and overlapping symptomatology between adjacent stages—particularly EMCI versus LMCI—severely limits the sensitivity of conventional neuropsychological assessments [6,7], motivating neuroimaging-based approaches for early pathological detection.

Resting-state functional magnetic resonance imaging (rs-fMRI), which measures the blood oxygen level-dependent (BOLD) signal during rest, has emerged as a particularly informative modality for probing brain-wide functional organization in AD [8]. By characterizing synchronous neural activity patterns across spatially distributed regions, rs-fMRI reveals functional connectivity (FC) disruptions that precede structural atrophy and cognitive symptom onset [9]. Notably, the default mode network (DMN), a set of regions preferentially active during rest, exhibits characteristic hypo-connectivity in AD patients compared to healthy controls, providing a sensitive and non-invasive imaging biomarker [10].

Traditional FC analyses assume stationarity—that connectivity patterns remain constant throughout the scanning session. However, converging evidence from neuroimaging studies indicates that brain functional connectivity fluctuates meaningfully over time, and that these dynamic fluctuations carry disease-relevant information beyond static summaries [11,12]. Dynamic functional connectivity (dFC), typically constructed via sliding-window Pearson correlation, captures this temporal variability by generating a sequence of connectivity matrices, each representing brain-network interactions within a short temporal window [13]. Studies have demonstrated that dFC analysis is more sensitive than static FC for detecting early-stage AD pathology, particularly in distinguishing EMCI from CN subjects where pathological changes are subtle and transient [14].

Despite its appeal, dFC-based AD classification faces three key technical challenges. First, each dFC window of a 116-region atlas yields a 6670-dimensional upper-triangular vector, creating a severe curse of dimensionality that demands effective nonlinear dimensionality reduction [14]. Second, the temporal ordering of dFC windows encodes trajectory information about brain state transitions, yet most methods discard this structure via simple pooling, or use sequential models (e.g., LSTM) that struggle with long-range dependencies [15,16]. Third, clinically acceptable models must be interpretable—a diagnosis without an explanation is difficult to validate or trust [17].

To address the dimensionality challenge, Variational Autoencoders (VAEs) [18] have been proposed as an unsupervised dimensionality reduction technique for dFC features. By learning a smooth, low-dimensional latent space constrained by KL divergence regularization, VAEs extract robust, noise-resistant representations that capture nonlinear connectivity patterns inaccessible to linear methods such as PCA [15].

For temporal sequence modeling, the recently proposed Mamba architecture [19]—a Selective State Space Model (S6)—offers a compelling alternative to recurrent neural networks and Transformer-based [20] attention mechanisms. Mamba introduces input-dependent selectivity: the transition matrices A, B, C, and the time-step Δ are dynamically computed from the input, enabling the model to selectively retain or discard information based on content relevance. Crucially, Mamba achieves linear computational complexity with respect to sequence length—in contrast to Transformer’s quadratic self-attention—making it particularly suitable for long sequences of dFC windows. While Mamba has demonstrated remarkable performance in natural language processing and genomics, its application to fMRI temporal dynamics for disease classification represents a significant and underexplored opportunity.

For interpretable classification, Kolmogorov–Arnold Networks (KAN) [21]—inspired by the Kolmogorov–Arnold representation theorem—replace the fixed node activations of MLPs with learnable univariate B-spline functions on edges. Each edge function $\varphi_{l,j,i}\left(x\right)$ is independently parameterized and directly visualizable, encoding the contribution of each latent feature to each class logit as an explicit, inspectable curve. This is fundamentally different from post hoc methods such as SHAP, which approximate feature contributions externally and incur additional computational cost. KAN’s intrinsic transparency is particularly valuable in clinical settings where the mechanism of a prediction matters as much as its accuracy.

In this paper, we propose **MambaKAN**, a unified, end-to-end interpretable framework that integrates VAE, Mamba, and KAN to address the aforementioned limitations synergistically. Specifically, the following are proposed:

1. A **VAE-based dynamic window encoder** maps each dFC snapshot independently into a compact 128-dimensional latent vector, effectively reducing the per-window feature dimension from 6670 to 128 while preserving nonlinear connectivity structure and suppressing noise via KL regularization.
2. A **Mamba temporal encoder** processes the sequence of 54 latent vectors (1 per dFC window) using selective state space dynamics, learning which temporal windows are most diagnostically relevant and capturing long-range dependencies across the entire scanning session with linear computational cost.
3. A **KAN classifier** maps the temporal context vector to diagnostic class probabilities through learnable B-spline activations, providing fully transparent, intrinsic interpretability without any post hoc approximation.
4. An **end-to-end joint training strategy** with differential learning rates ensures that VAE features are refined toward classification objectives while the Mamba and KAN modules learn effectively from a rich, pre-trained latent space.

The main contributions of this work are as follows:

1. This work represents one of the pioneering efforts to apply a Selective State Space Model (Mamba) to the modeling of temporal dynamics in dFC for AD classification, demonstrating superior performance over LSTM and Transformer-based alternatives in capturing clinically relevant brain state trajectories.
2. We integrate KAN as the classification backbone, providing intrinsic interpretability through visualizable activation functions. This enables direct inspection of how each latent dimension influences classification decisions, without relying on post hoc approximation methods.
3. We design a principled two-phase joint training strategy with a composite loss function that tightly couples reconstruction fidelity and classification accuracy, ensuring that feature learning is task-aligned and yielding representations that outperform unsupervised pre-training followed by fixed feature extraction.
4. We provide a multi-layered interpretability analysis that combines Mamba’s temporal selectivity scores with KAN activation curve visualization and gradient-based brain region attribution, offering complementary neuroscientific insights at the temporal, functional, and anatomical levels.
5. We conduct comprehensive experiments across five clinically relevant classification tasks on the ADNI dataset, including ablation studies and sensitivity analyses, demonstrating consistent improvements over seven competitive baselines.

The remainder of this paper is organized as follows. Section 2 describes the ADNI dataset and preprocessing pipeline. Section 3 presents the complete MambaKAN architecture and training procedure. Section 4 reports experimental results, baseline comparisons, and ablation studies. Section 5 presents the multi-layered interpretability analysis. Section 6 analyzes the computational complexity and inference latency of all compared models. Section 7 discusses findings, limitations, and future directions. Section 8 concludes the paper.

## 2. Materials

### 2.1. Dataset Description

In this study, we utilized rs-fMRI data from the Alzheimer’s Disease Neuroimaging Initiative (ADNI) dataset [22,23]. ADNI is a large-scale, multi-center, longitudinal study designed to advance the understanding of AD pathogenesis and progression through the integration of neuroimaging, biomarker profiling, and cognitive assessment. The dataset comprises 174 unique subjects representing four diagnostic categories: 48 cognitively normal (CN), 50 with early mild cognitive impairment (EMCI), 45 with late mild cognitive impairment (LMCI), and 31 with AD. Each subject contributed one or more longitudinal scans, yielding a total of 563 scan sessions distributed as follows: 154 CN, 165 EMCI, 145 LMCI, and 99 AD. All scans were acquired on 3.0T MRI scanners (Philips Healthcare, Best, The Netherlands; Siemens Healthineers, Erlangen, Germany; GE Healthcare, Chicago, IL, USA) across multiple medical centers under standardized imaging parameters: flip angle, 80°; image resolution, 2.29–3.31 mm; slice thickness, 3.31 mm; echo time (TE) = 30 ms; repetition time (TR) range, 2.2–3.1 s; imaging matrix, 64×64 pixels; field of view, 256×256 mm^2^; total scanning duration, 7 min, encompassing 140 image volumes.

### 2.2. Data Preprocessing

The preprocessing pipeline for rs-fMRI data follows established protocols adapted from related work [15,16,19]. Standard preprocessing was performed using FSL FEAT software (v6.0, FMRIB, University of Oxford, Oxford, UK), comprising the following sequential steps: (i) discarding the first three volumes to allow longitudinal magnetization equilibration; (ii) slice-timing correction to account for temporal acquisition offsets between slices; (iii) head motion correction using rigid-body registration; (iv) band-pass temporal filtering (0.01–0.08 Hz) to isolate low-frequency BOLD fluctuations; and (v) nuisance covariate regression including white matter signal, cerebrospinal fluid signal, global signal, and the six motion parameters. Subjects exhibiting head motion exceeding 2 mm translational displacement or 2° rotational displacement were excluded from analysis to ensure data quality.

Structural preprocessing involved skull stripping from T1-weighted sMRI images, followed by nonlinear registration to Montreal Neurological Institute (MNI) standard space. Functional images were subsequently smoothed using a 6 mm full-width at half-maximum (FWHM) isotropic Gaussian kernel to improve the signal-to-noise ratio. Regional parcellation was performed using the Automated Anatomical Labeling (AAL) atlas, which partitions the brain into 116 anatomically defined regions of interest (ROIs). The mean BOLD time series was extracted for each ROI, yielding a subject-level data matrix $X\in R^{116\times T}$, where *T* denotes the number of time points following preprocessing. Individual ROI time series were *z*-score normalized across time:

(1) $$x_{i,\mathrm{norm}}\left(t\right)=\frac{x_{i}\left(t\right)-\mu_{i}}{\sigma_{i}},$$

where $\mu_{i}$ and $\sigma_{i}$ denote the temporal mean and standard deviation of the *i*-th ROI time series, respectively.

### 2.3. Dynamic Functional Connectivity Construction

To capture the time-varying nature of brain functional interactions, we constructed dFC matrices using a sliding-window approach. The normalized ROI time series of each subject was segmented into overlapping sub-windows of a length of $L_{w}=30$ time points (corresponding to approximately 66–90 s depending on TR) with a step size of s=2 time points (approximately 4.4–6.2 s). This parameterization balances temporal resolution against signal stability: shorter windows risk statistical instability in correlation estimation, while longer windows smooth out genuine dynamic fluctuations [13]. The resulting window count per subject is

(2) $$K=\left\lfloor \frac{T-L_{w}}{s}\right\rfloor +1=54,$$

yielding K=54 overlapping windows per subject. For the *k*-th window $S_{k}\in R^{116\times 30}$, the dFC matrix $R_{k}\in R^{116\times 116}$ is computed via pairwise Pearson correlation:

(3) $$r_{k,ij}=\frac{\sum_{t=1}^{L_{w}}\left(x_{i}^{\left(k\right)}\left(t\right)-{\bar{x}}_{i}^{\left(k\right)}\right)\left(x_{j}^{\left(k\right)}\left(t\right)-{\bar{x}}_{j}^{\left(k\right)}\right)}{\sqrt{\sum_{t=1}^{L_{w}}{\left(x_{i}^{\left(k\right)}\left(t\right)-{\bar{x}}_{i}^{\left(k\right)}\right)}^{2}}\cdot \sqrt{\sum_{t=1}^{L_{w}}{\left(x_{j}^{\left(k\right)}\left(t\right)-{\bar{x}}_{j}^{\left(k\right)}\right)}^{2}}},$$

where $x_{i}^{\left(k\right)}\left(t\right)$ is the BOLD signal of the *i*-th ROI at the *t*-th time point within window *k*, and ${\bar{x}}_{i}^{\left(k\right)}$ is its within-window mean. Since $R_{k}$ is symmetric with a unit diagonal, we extract only the upper-triangular off-diagonal elements to form a non-redundant feature vector. For an atlas with N=116 ROIs, this yields N(N−1)/2=6670 features per window. The vectorized dFC representation for window *k* is denoted as $v_{k}\in R^{6670}$. The full dFC sequence for a subject is thus ${\left\{v_{k}\right\}}_{k=1}^{54}\in R^{54\times 6670}$.

### 2.4. Dataset Partitioning

To prevent data leakage, all dataset splits were performed at the subject level: all scan sessions of the same subject are assigned exclusively to one partition. We employ a fixed 70/10/20 (train/validation/test) split with pre-determined indices saved as JSON files for full reproducibility. The 174 subjects are distributed across the four diagnostic categories as follows: 48 CN, 50 EMCI, 45 LMCI, and 31 AD. The fixed partitioning maintains approximate class balance across training, validation, and test sets, with the smallest class (AD) represented in all three partitions to ensure reliable performance evaluation. Table 1 provides the per-class subject count in each partition, offering full transparency over the distribution of the smallest diagnostic group.

Classification tasks are defined as five settings: (T1) CN vs. AD, (T2) CN vs. EMCI, (T3) EMCI vs. LMCI, (T4) LMCI vs. AD, and (T5) four-class (CN/EMCI/LMCI/AD). All classification metrics are computed at the subject level: the 54 window-level predictions for each subject are aggregated via probability averaging to produce a single subject-level label, which is then compared against the ground truth. This ensures that the high temporal autocorrelation between adjacent windows (step size s=2 yields $\frac{L_{w}-s}{L_{w}}=93\%$ overlap) does not inflate performance estimates.

## 3. Methods

### 3.1. Overview of MambaKAN

**Figure 1:**
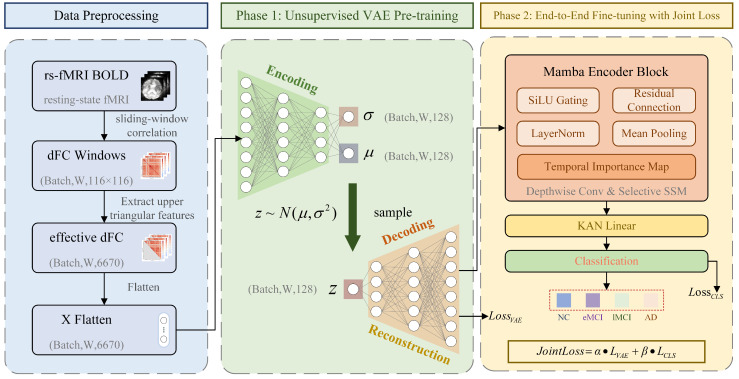
Overall architecture of the proposed MambaKAN framework. The pipeline sequentially transforms rs-fMRI BOLD signals through dFC construction, VAE-based per-window latent encoding, Mamba selective temporal modeling, and KAN-based interpretable classification: dynamic functional connectivity construction via sliding-window Pearson correlation; Variational Autoencoder per-window encoding with reparameterization; Mamba selective state space temporal encoder aggregating the 54-window latent sequence; KAN classifier with learnable B-spline edge activations producing class probabilities and interpretable activation curves.

Figure 1 illustrates the MambaKAN architecture, comprising four sequential stages: (1) dFC construction from rs-fMRI BOLD time series; (2) per-window VAE encoding of each dFC snapshot into a 128-dimensional latent vector; (3) Mamba temporal modeling over the 54-element latent sequence; and (4) KAN classification with intrinsically interpretable B-spline activations. The pipeline is trained end-to-end via a joint objective balancing reconstruction and classification.

### 3.2. Variational Autoencoder for Per-Window Feature Extraction

#### 3.2.1. Encoder Architecture

The VAE encoder $q_{\phi }\left(z|v\right)$ maps each 6,670-dimensional dFC window vector $v_{k}$ to a posterior distribution over a 128-dimensional latent space. The encoder is implemented as a fully connected multi-layer network with progressive dimensionality reduction:

(4) $$h^{\left(1\right)}=\mathrm{ReLU}\left(W_{1}v+b_{1}\right),\;h^{\left(1\right)}\in R^{2048},$$

(5) $$h^{\left(\ell \right)}=\mathrm{ReLU}\left(W_{\ell }h^{\left(\ell -1\right)}+b_{\ell }\right),\;\ell =2,3,4,$$

with hidden dimensions progressing as [6670→2048→1024→512→256]. The final hidden layer feeds two parallel linear projections producing the following variational parameters:

(6) $$\mu_{z}=W_{\mu }h^{\left(4\right)}+b_{\mu },\;\log\sigma_{z}^{2}=W_{\sigma }h^{\left(4\right)}+b_{\sigma },\;\mu_{z},\log\sigma_{z}^{2}\in R^{128}.$$

The latent variable $z\in R^{128}$ is sampled via the reparameterization trick to allow gradient-based optimization:

(7) $$z=\mu_{z}+\sigma_{z}⊙\epsilon ,\;\epsilon ∼N\left(0,I\right),$$

where ⊙ denotes element-wise multiplication.

#### 3.2.2. Decoder Architecture

The decoder $p_{\theta }\left(v|z\right)$ reconstructs the input dFC vector from the latent sample using a mirror-symmetric fully connected network with dimensions [128→256→512→1024→2048→6670]. Intermediate layers use ReLU activations, while the final layer employs a Sigmoid function to constrain reconstructed values to [0, 1], consistent with the normalized correlation coefficient range:

(8) $$\hat{v}=\sigma \left(W_{\mathrm{dec}}^{\left(5\right)}h_{\mathrm{dec}}^{\left(4\right)}+b_{\mathrm{dec}}^{\left(5\right)}\right),$$

where σ(·) is the sigmoid activation.

#### 3.2.3. VAE Loss Function

The VAE is trained to minimize the evidence lower bound (ELBO), which decomposes into a reconstruction term and a KL divergence regularization term:

(9) $$L_{\mathrm{VAE}}=L_{\mathrm{recon}}+\beta \;L_{\mathrm{KL}},$$

where the reconstruction loss measures mean squared error between the input and its reconstruction:

(10) $$L_{\mathrm{recon}}=\frac{1}{N_{b}}\sum_{n=1}^{N_{b}}{∥v_{n}-{\hat{v}}_{n}∥}_{2}^{2},$$

and the KL divergence constrains the approximate posterior $q_{\phi }\left(z|v\right)$ toward the standard normal prior p(z)=N(0,I):

(11) $$L_{\mathrm{KL}}=-\frac{1}{2}\sum_{j=1}^{L}\left(1+\log\sigma_{z,j}^{2}-\mu_{z,j}^{2}-\sigma_{z,j}^{2}\right),$$

with L=128 denoting the latent dimension and β=1.0. The KL term regularizes the latent space toward a smooth normal distribution, suppressing noise from head motion artifacts, physiological signals, and scanner instabilities prevalent in rs-fMRI data.

#### 3.2.4. Window-Level Independent Encoding

Each of the 54 dFC windows is encoded independently, yielding a latent sequence $Z=\left[z_{1},\ldots ,z_{54}\right]\in R^{54\times 128}$. This preserves per-window temporal identity—global averaging would conflate distinct brain states—and provides the ordered sequence required by the Mamba encoder.

### 3.3. Mamba Selective State Space Temporal Encoder

#### 3.3.1. Rationale for Mamba over LSTM and Transformer

The choice of Mamba as the temporal encoder is motivated by three key limitations of conventional sequence models in the context of dFC analysis. First, Long Short-Term Memory (LSTM) networks [24], while effective for short-to-medium sequences, suffer from the vanishing gradient problem and limited context window when processing long sequences: the 54-window dFC trajectory spans the entire 7-min scanning session, and LSTM’s recurrent bottleneck struggles to propagate information across such extended temporal horizons without degradation. Second, Transformer architectures [20], despite their ability to model global dependencies via self-attention, incur quadratic computational complexity $O(L^{2}d)$ with respect to sequence length *L*, making them parameter-inefficient and memory-intensive for long sequences—a critical concern given the small sample size of medical imaging datasets where model capacity must be carefully allocated. Third, neither LSTM nor standard Transformer incorporates content-based selectivity: they process all time steps uniformly, whereas dFC sequences contain both diagnostically informative windows (e.g., early-scan stable states) and noisy or artifact-contaminated windows that should be down-weighted.

Mamba addresses all three limitations simultaneously. Its Selective State Space Model (S6) mechanism achieves linear computational complexity O(Ld) through hardware-aware parallel scan algorithms, enabling efficient processing of long dFC sequences without the quadratic cost of self-attention. More importantly, Mamba’s input-dependent selectivity—where the state transition matrices $B_{k}$, $C_{k}$ and time-step $\Delta_{k}$ are dynamically computed from the input $z_{k}$—this allows the model to adaptively filter the temporal stream: large $\Delta_{k}$ values cause the model to “forget” prior context and focus on the current window (high selectivity), while small $\Delta_{k}$ values preserve long-range dependencies (low selectivity). This content-aware gating is particularly well-suited to dFC data, where the diagnostic relevance of each time window varies based on brain state dynamics, head motion artifacts, and subject-specific scanning conditions. Empirical validation confirms that Mamba (70.5% accuracy) outperforms both BiLSTM (68.2%) and mean pooling (59.4%) on the four-class task, demonstrating that selective temporal modeling provides a measurable advantage over uniform aggregation or recurrent processing.

#### 3.3.2. State Space Model Foundations

Continuous-time linear state space models (SSMs) describe input–output dynamics via a hidden state $h\left(t\right)\in R^{N}$:

(12) $$\dot{h}\left(t\right)=Ah\left(t\right)+Bu\left(t\right),\;y\left(t\right)=Ch\left(t\right),$$

where $A\in R^{N\times N}$, $B\in R^{N\times 1}$, $C\in R^{1\times N}$ are learnable parameters, u(t) is the scalar input, and y(t) is the scalar output. Discretization with step size Δ via zero-order hold yields

(13) $$\bar{A}=e^{\Delta A},\;\bar{B}={\left(\Delta A\right)}^{-1}\left(e^{\Delta A}-I\right)\Delta B,$$

so that the discrete recurrence becomes the following:

(14) $$h_{k}=\bar{A}h_{k-1}+\bar{B}u_{k},\;y_{k}=Ch_{k}.$$

#### 3.3.3. Selective State Space Model (S6)

The Mamba architecture [19] extends the SSM to a Selective State Space Model (S6) by making the parameters B, C, and the time-step Δ input-dependent:

(15) $$B_{k}=B\left(z_{k}\right),\;C_{k}=C\left(z_{k}\right),\;\Delta_{k}=\mathrm{softplus}\left(\Delta (z_{k})\right),$$

where B(·), C(·), and Δ(·) are lightweight linear projections. This selectivity mechanism allows the model to filter the input stream based on content: large $\Delta_{k}$ values cause ${\bar{A}}_{k}\approx 0$ (the state “forgets” and focuses on the current input), while small $\Delta_{k}$ values cause ${\bar{A}}_{k}\approx I$ (the state carries forward prior context). For multi-dimensional inputs $z_{k}\in R^{d_{\mathrm{model}}}$, the S6 operation is applied independently per dimension, and the full Mamba block incorporates a depthwise convolution branch and gated multiplicative output, as detailed below.

#### 3.3.4. Mamba Block Architecture

Each Mamba block processes the input sequence $Z\in R^{54\times d_{\mathrm{model}}}$ with $d_{\mathrm{model}}=128$ through the following operations:

(16) $$Z^{\prime }=\mathrm{LayerNorm}\left(Z\right),$$

(17) $$X=Z^{\prime }W_{x}\in R^{54\times d_{\mathrm{inner}}},\;G=\mathrm{SiLU}\left(Z^{\prime }W_{g}\right)\in R^{54\times d_{\mathrm{inner}}},$$

(18) $$X^{\prime }=\mathrm{DepthwiseConv}1d\left(X\right),\;X^{\prime \prime }=\mathrm{SiLU}\left(X^{\prime }\right),$$

(19) $$Y=S6\left(X^{\prime \prime }\right),\;Y^{\prime }=Y⊙G,$$

(20) $$\mathrm{Output}=Z+Y^{\prime }W_{\mathrm{out}},$$

where $d_{\mathrm{inner}}=2\times d_{\mathrm{model}}=256$ (expansion factor E=2), the depthwise convolution has a kernel size of $d_{\mathrm{conv}}=4$, and the SiLU (Sigmoid Linear Unit) denotes SiLU(x)=x·σ(x). The residual connection ensures gradient flow during deep network training. We stack $L_{m}=2$ Mamba blocks, as empirically determined via ablation.

The S6 sub-module computes the following for each dimension $d\in \{1,\ldots ,d_{\mathrm{inner}}\}$:

(21) $$\Delta_{k,d}=\mathrm{softplus}\left(W_{\Delta }^{\left(d\right)}x_{k,d}^{\prime \prime }\right),$$

(22) $${\bar{A}}_{k,d}=\exp\left(\Delta_{k,d}\cdot A_{d}\right),$$

(23) $${\bar{B}}_{k,d}=\Delta_{k,d}\cdot B_{k,d},\;B_{k,d}=W_{B}^{\left(d\right)}z_{k},$$

(24) $$h_{k,d}={\bar{A}}_{k,d}\cdot h_{k-1,d}+{\bar{B}}_{k,d}\cdot x_{k,d}^{\prime \prime },$$

(25) $$y_{k,d}=C_{k,d}\cdot h_{k,d},\;C_{k,d}=W_{C}^{\left(d\right)}z_{k},$$

where $A_{d}\in R^{d_{\mathrm{state}}}$ is a learnable structured state matrix initialized with HiPPO [19], and $d_{\mathrm{state}}=16$ is the internal SSM state dimension.

#### 3.3.5. Temporal Context Aggregation

After processing through $L_{m}=2$ stacked Mamba blocks, the output sequence $O\in R^{54\times 128}$ is aggregated into a single temporal context vector via mean pooling:

(26) $$c=\frac{1}{K}\sum_{k=1}^{K}O_{k}\in R^{128}.$$

Mean pooling provides a stable, permutation-aware aggregation that summarizes all temporal positions equally after selective attention has already emphasized the most discriminative windows through the $\Delta_{k}$ gating mechanism.

#### 3.3.6. Temporal Interpretability via Selectivity Scores

The per-step time-step values ${\left\{\Delta_{k}\right\}}_{k=1}^{54}$ provide a natural measure of temporal importance: a large $\Delta_{k}$ indicates that the model assigns high relevance to window *k* (fresh information is prioritized), while a small $\Delta_{k}$ indicates that the prior state is carried forward with minimal update from window *k*. We define the temporal importance score for window *k* as follows:

(27) $$s_{k}=\frac{1}{d_{\mathrm{inner}}}\sum_{d=1}^{d_{\mathrm{inner}}}\Delta_{k,d},$$

averaged across all inner dimensions to produce a scalar summary. These scores can be aggregated across subjects within each diagnostic class to reveal the class-specific temporal patterns of brain state dynamics.

### 3.4. Kolmogorov–Arnold Network Classifier

#### 3.4.1. KAN Architecture Motivation

Standard MLPs parameterize activation functions (e.g., ReLU, GELU) as fixed nonlinearities on nodes, while the learnable weights reside on edges. Kolmogorov–Arnold Networks (KAN) [21], inspired by the Kolmogorov–Arnold representation theorem, which states that any continuous multivariate function $f:{\left[0,1\right]}^{n}\to R$ can be written as a finite composition of univariate functions and addition, instead place learnable univariate functions on edges, with each edge representing an independently parameterized transformation. This architectural choice enables the network to be intrinsically interpretable: each edge function $\varphi_{l,j,i}$ directly encodes the contribution of the *i*-th unit of layer *l* to the *j*-th unit of layer l+1 as a visualizable curve, independent of all other edges.

#### 3.4.2. Rationale for KAN over MLP

The choice of KAN as the classification backbone is motivated by two critical requirements of medical imaging analysis: parameter efficiency and intrinsic interpretability. First, standard MLPs with ReLU or GELU activations require large hidden layers to approximate complex nonlinear decision boundaries, leading to parameter proliferation that exacerbates overfitting on small medical datasets. In contrast, KAN parameterizes each edge connection as an independent B-spline function, enabling the network to learn smooth, nonlinear transformations with far fewer parameters: replacing the MLP classification head with KAN adds only ∼43 K parameters (+0.13%) while improving four-class accuracy from 62.5% to 70.5%. The B-spline basis functions inherently encode smoothness priors through their piecewise polynomial structure, providing implicit regularization that is particularly beneficial when training on the limited ADNI cohort (n=174 subjects).

Second, and more fundamentally, KAN provides intrinsic interpretability that is qualitatively different from post hoc explanation methods. In a standard MLP, the contribution of a latent feature to the final prediction is mediated through multiple layers of fixed nonlinear activations (e.g., ReLU) and linear projections, making it impossible to visualize the feature-to-prediction mapping without external approximation tools like SHAP or LIME. These post hoc methods compute local linear approximations around specific test samples and require additional forward passes, yielding explanations that are sample-dependent and computationally expensive. In contrast, KAN’s edge activation functions $\varphi_{j,i}\left(x\right)$ are model parameters that can be directly plotted after training, revealing the global, nonlinear relationship between each latent dimension and the classification logits without any post hoc computation. This transparency is critical for clinical trust and regulatory approval: a radiologist can inspect the learned activation curves and verify that the model’s decision logic aligns with known pathophysiology, rather than relying on black-box predictions justified only by aggregate accuracy metrics.

#### 3.4.3. B-Spline Edge Activations

Each edge activation φ(x):R→R is parameterized as a sum of a scaled residual SiLU function and a learnable B-spline:

(28) $$\varphi \left(x\right)=w_{b}\cdot \mathrm{SiLU}\left(x\right)+\sum_{i=1}^{G+p}c_{i}\cdot B_{i,p}\left(x\right),$$

where $w_{b}\in R$ is a learnable base weight, $c_{i}\in R$ are B-spline coefficients, $B_{i,p}\left(x\right)$ are B-spline basis functions of order p=3 (cubic) defined on a uniform grid of G=5 intervals over the input domain $\left[x_{\min},x_{\max}\right]$. The total number of parameters per edge is G+p=8. During training, the grid is periodically updated to span the current activation range, preventing numerical instability from out-of-range inputs.

#### 3.4.4. KAN Layer Forward Pass

A KAN layer with $n_{\mathrm{in}}$ input neurons and $n_{\mathrm{out}}$ output neurons computes the following:

(29) $$y_{j}=\sum_{i=1}^{n_{\mathrm{in}}}\varphi_{j,i}\left(x_{i}\right),\;j=1,\ldots ,n_{\mathrm{out}},$$

where $\varphi_{j,i}$ is a distinct B-spline activation parameterized as in Equation (28).

#### 3.4.5. MambaKAN Classifier Structure

The KAN classifier receives the temporal context vector $c\in R^{128}$ from the Mamba encoder and produces class logits $\hat{y}\in R^{C}$ through a two-layer KAN:

(30) $$\begin{array}{cc} h_{\mathrm{KAN}} & ={\mathrm{KAN}}_{1}\left(c;\left\{\varphi_{j,i}^{\left(1\right)}\right\}\right)\in R^{64}, \end{array}$$

(31) $$\begin{array}{cc} \hat{y} & ={\mathrm{KAN}}_{2}\left(h_{\mathrm{KAN}};\left\{\varphi_{j,i}^{\left(2\right)}\right\}\right)\in R^{C}, \end{array}$$

where *C* is the number of classes (two for binary tasks, four for the four-class task). The total number of learnable activation parameters in the KAN classifier is (128×64+64×C)×8 spline coefficients plus 128×64+64×C base weights.

#### 3.4.6. KAN Interpretability

Following training, each activation curve $\varphi_{j,i}^{\left(l\right)}$ can be directly plotted as a function of its input, revealing the nature of the relationship between the *i*-th latent dimension (or hidden unit) and the *j*-th downstream unit. Monotonically increasing curves indicate positive linear-like contributions; non-monotonic curves reveal threshold effects or u-shaped relationships; flat curves indicate that a particular connection has been effectively pruned. This intrinsic transparency eliminates the need for post hoc attribution methods like SHAP or LIME, which provide only approximate local explanations and require additional computation after training.

### 3.5. Joint Training Strategy

#### 3.5.1. Two-Phase Training Protocol

To ensure stable and effective learning, we employ a two-phase training strategy that exploits the complementary objectives of unsupervised representation learning and supervised classification:

Phase 1—VAE Unsupervised Pre-training:The VAE is first trained in isolation on the full training set (without labels) to minimize $L_{\mathrm{VAE}}$ (Equation (9)). This phase runs for 100 epochs with the Adam optimizer at a learning rate of $\eta_{1}=10^{-3}$ and a batch size of 32. By pre-training without labels, the VAE learns a general-purpose, noise-robust latent representation of dFC dynamics that is not biased toward any specific classification task, improving downstream generalization. The best checkpoint (lowest validation reconstruction loss) is saved for Phase 2 initialization.

Phase 2—End-to-End Joint Fine-tuning: The full MambaKAN pipeline (VAE + Mamba + KAN) is trained jointly using the composite loss:

(32) $$L_{\mathrm{total}}=\alpha \;L_{\mathrm{VAE}}+\beta \;L_{\mathrm{cls}},$$

where $L_{\mathrm{cls}}$ is the cross-entropy classification loss:

(33) $$L_{\mathrm{cls}}=-\frac{1}{N_{b}}\sum_{n=1}^{N_{b}}\sum_{c=1}^{C}y_{n,c}\log{\hat{p}}_{n,c},$$

with $y_{n,c}$ the one-hot label and ${\hat{p}}_{n,c}$ the Softmax probability for sample *n* and class *c*. We set α=0.1 and β=1.0, treating reconstruction as a regularizer that prevents the catastrophic forgetting of the VAE’s learned manifold structure, while prioritizing classification accuracy.

#### 3.5.2. Differential Learning Rates

To protect the pre-trained VAE representations from destructive updates, we apply differential learning rates in Phase 2:

(34) $$\eta_{\mathrm{VAE}}=10^{-5},\;\eta_{\mathrm{Mamba}}=\eta_{\mathrm{KAN}}=10^{-3}.$$

The Mamba and KAN parameters use a 100× larger learning rate, allowing rapid adaptation while the VAE undergoes only fine-grained updates. During the initial $E_{\mathrm{warm}}=15$ warmup epochs of Phase 2, VAE parameters are frozen to stabilize Mamba and KAN before joint fine-tuning begins.

#### 3.5.3. Regularization

To mitigate overfitting on the small ADNI dataset, Dropout (p=0.15) is applied within each Mamba block after the depthwise convolution. The VAE KL divergence (Equation (11)) additionally regularizes the latent space. No Dropout is applied within KAN layers to preserve interpretability of individual activation curves.

#### 3.5.4. Optimization Details

Phase 2 training runs for 100 epochs with a batch size of 32. The Adam optimizer is used with $\beta_{1}=0.9$, $\beta_{2}=0.999$, $\epsilon =10^{-8}$. The best checkpoint (highest validation accuracy) is saved for final evaluation. All experiments are implemented in PyTorch 2.0 on an NVIDIA GPU with 16 GB VRAM.

## 4. Experiments

### 4.1. Implementation Details

The MambaKAN framework was implemented in PyTorch 2.0 with Python 3.8. The VAE architecture follows the design described in Section 3.2 with encoder dimensions [6670, 2048, 1024, 512, 256, 128] and symmetric decoder. The Mamba encoder uses $L_{m}=2$ layers, $d_{\mathrm{model}}=128$, $d_{\mathrm{state}}=16$, $d_{\mathrm{conv}}=4$, an expansion factor of E=2, and a Dropout rate of 0.15. The KAN classifier has two layers with dimensions [128→64→C], a B-spline grid size of G=5, and a spline order of p=3. To assess the statistical stability of all reported results, each experiment was repeated with five fixed random seeds on the same hardware under the fixed 70/10/20 subject-level train/validation/test split described in Section 2. Performance metrics are reported as mean ± standard deviation across these five runs.

### 4.2. Evaluation Metrics

Classification performance is evaluated using four standard metrics: accuracy (Acc), macro-averaged precision (Pre), macro-averaged recall (Rec), and macro-averaged F1-score (F1). For binary tasks, the area under the ROC curve (AUC) is additionally reported. Statistical significance of performance differences between MambaKAN and each baseline was assessed using a paired *t*-test (α=0.05) on run-level accuracy scores across the five seeds.

### 4.3. Baseline Methods

We compare MambaKAN against the following seven baselines:

- **VAE + MLP:** A VAE encoder for per-window feature extraction followed by mean pooling and a hierarchical MLP classifier, without temporal modeling or intrinsic interpretability.
- **VAE + BiLSTM:** Mamba replaced by a two-layer bidirectional LSTM [24] (hidden size of 128 per direction).
- **VAE + Transformer:** Mamba replaced by a two-layer Transformer encoder [20] (four heads, a feedforward dimension of 256, and sinusoidal positional encoding).
- **MambaKAN (no pre-training):** Full MambaKAN trained from random initialization, without Phase 1 VAE pre-training.
- **VAE + KAN (no Mamba):** Mamba replaced by mean pooling over the 54 latent vectors, feeding directly into the KAN classifier.
- **VAE + Mamba + MLP (no KAN):** KAN replaced by a standard two-layer MLP (hidden size 64, ReLU).
- **Traditional ML:** SVM-RBF, Random Forest, and Gradient Boosting on mean-pooled VAE latent features.

### 4.4. Classification Performance Comparison

Table 2 and Table 3 present the classification performance of MambaKAN and all seven baseline methods across the five tasks on the ADNI dataset.

### 4.5. Ablation Study

Table 4 presents the ablation study on the four-class task (T5), analyzing the contribution of each major component.

### 4.6. Sensitivity Analysis

Table 5 examines the sensitivity of MambaKAN to key hyperparameters on the CN vs. AD task (T1).

## 5. Interpretability Analysis

A central contribution of MambaKAN is its multi-layer interpretability framework, operating simultaneously at three complementary levels: temporal (Mamba), functional (KAN), and anatomical (gradient attribution). This hierarchical analysis provides insights inaccessible to single-level post hoc methods.

### 5.1. Layer 1: Mamba Temporal Importance

**Figure 2:**
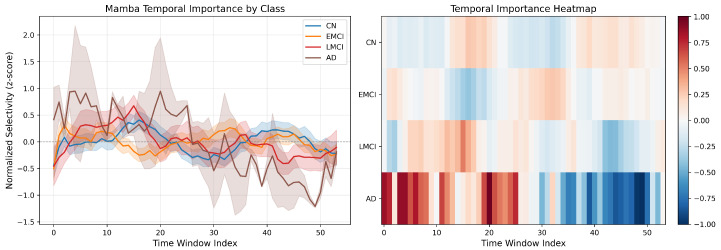
Mamba temporal importance profiles by diagnostic class. The *y*-axis shows the mean time-step selectivity score $s_{k}$ (Equation (27)) averaged over test subjects within each class, and the shaded regions indicate ±1 standard deviation. Elevated $s_{k}$ values at specific windows indicate that those temporal snapshots are most informative for discriminating the respective diagnostic class.

Using the temporal importance scores defined in Equation (27), we compute the mean $\Delta_{k}$ profile for each diagnostic class by averaging over all subjects in the test set. Figure 2 visualizes these profiles across the 54 dFC windows.

The analysis reveals class-specific temporal patterns consistent with known AD pathophysiology. The AD class (brown curve) exhibits a distinctive biphasic profile: significantly elevated selectivity during early time windows (0–25), with peak z-scores approaching +1.0, followed by a sharp decline to negative values (minimum ≈−1.0) in late windows (30–55). This pattern suggests that early-scan brain states contain the most discriminative AD signatures, while late-scan states actively contradict AD classification—potentially reflecting fatigue-related or attention-related signal degradation that disproportionately affects AD patients. In contrast, the CN, EMCI, and LMCI classes exhibit relatively flat selectivity profiles centered near zero, indicating temporally uniform feature distributions without pronounced critical windows. The LMCI class shows a subtle progressive increase across the scan, intermediate between the stable CN/EMCI profiles and the extreme AD biphasic pattern, consistent with LMCI’s position on the disease continuum. This temporal specificity—particularly the early-window AD peak—provides data-driven evidence for optimizing clinical scanning protocols: shorter, early-focused acquisitions may suffice for AD detection, reducing patient burden and scan costs.

### 5.2. Layer 2: KAN Activation Curve Analysis

**Figure 3:**
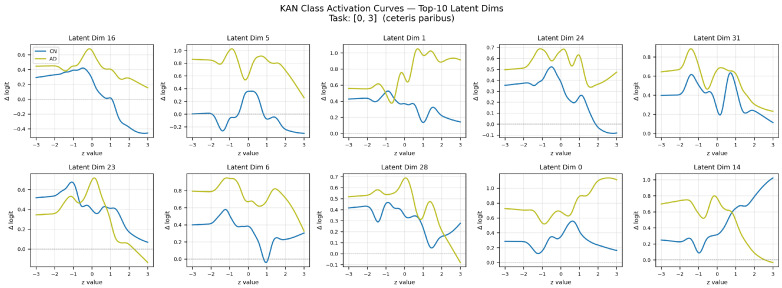
Learned KAN activation curves for the top-10 most influential latent dimensions (CN vs. AD task). Each panel shows the B-spline edge function $\varphi_{j,i}^{\left(1\right)}\left(x\right)$ as a function of the latent input value, with color indicating the corresponding class logit weight. Monotonically increasing (decreasing) curves indicate dimensions whose elevated values promote AD (CN) classification; non-monotonic curves reveal threshold or saturation effects in latent-to-class mappings.

Figure 3 presents the learned B-spline activation curves $\varphi_{j,i}^{\left(1\right)}$ for the top-K=10 most influential latent dimensions (ranked by mean absolute activation magnitude) in the first KAN layer for the CN vs. AD binary classification task.

The activation curves reveal three interpretable patterns. Certain latent dimensions exhibit monotonically increasing activation curves for the AD logit, indicating that their elevated values consistently promote AD classification; these likely encode persistent connectivity reduction in the DMN and hippocampal networks. Conversely, latent dimensions with monotonically decreasing curves correspond to features whose suppression is associated with AD pathology, potentially encoding preserved connectivity in frontal networks observed in early-stage patients. Non-monotonic curves (particularly sigmoidal or u-shaped) indicate a nonlinear contribution to classification—such dimensions may correspond to connectivity features that are pathological only outside a normative range. Critically, across all 10 latent dimensions, the AD class (yellow curves) consistently exhibits higher Δ logit values than the CN class (blue curves) without any rank reversals, demonstrating that KAN has learned stable, monotonic class-discriminative features rather than overfitting to noise.

**Figure 4:**
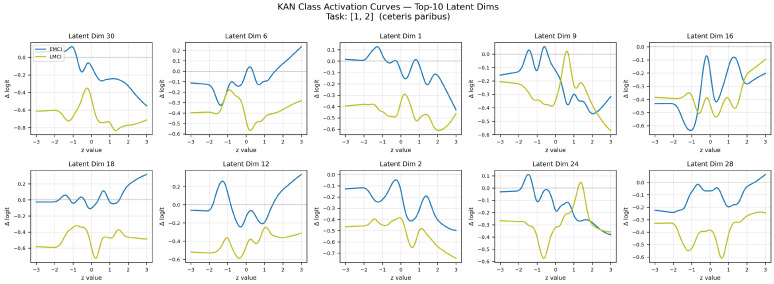
Learned KAN activation curves for the top-10 most influential latent dimensions (EMCI vs. LMCI task). Each panel shows the B-spline edge function $\varphi_{j,i}^{\left(1\right)}\left(x\right)$ as a function of the latent input value. The close proximity and frequent overlap of the two curves reflect the clinical reality that EMCI and LMCI represent adjacent stages on the AD continuum with highly overlapping pathological features, making this the most challenging binary classification task.

The EMCI vs. LMCI activation curves (Figure 4) present a striking contrast to the CN vs. AD case. While the EMCI class (blue) consistently maintains higher Δ logit values than LMCI (yellow) across all dimensions—preserving monotonic class ordering—the inter-class separation is markedly reduced, with curves frequently overlapping or running in close parallel. This compressed separation quantitatively reflects the clinical challenge of distinguishing adjacent MCI stages: EMCI and LMCI share substantial pathological overlap, differing primarily in severity rather than qualitative feature profiles. The smooth, continuous nature of all curves confirms that KAN captures biologically plausible nonlinear relationships rather than spurious discontinuities, even in this difficult discrimination regime.

### 5.3. Layer 3: Gradient-Based Brain Region Attribution

**Figure 5:**
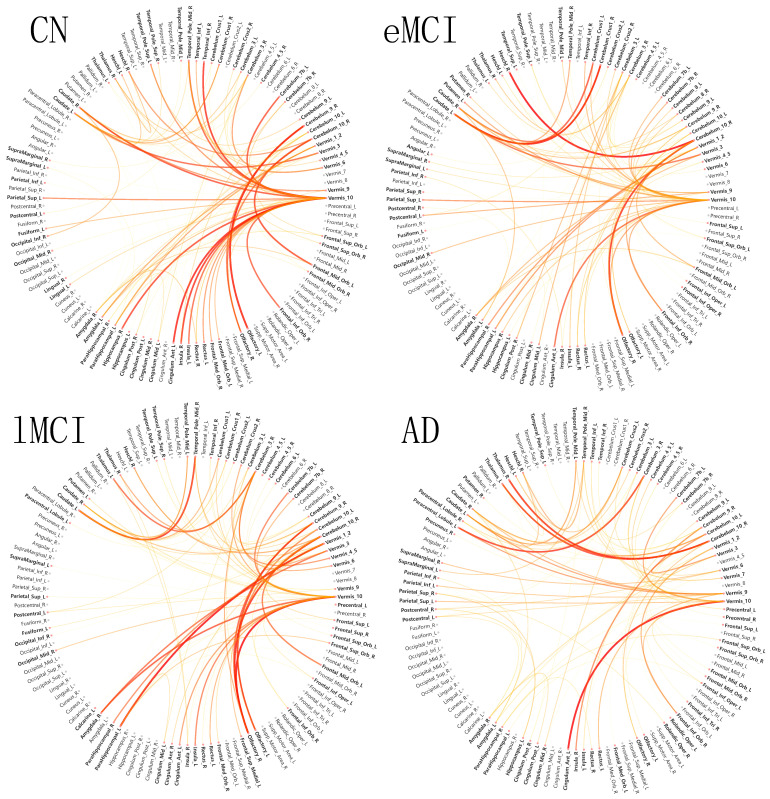
Gradient-based brain region attribution chord diagrams for each diagnostic class. Each panel visualizes the top-50 most influential ROI-to-ROI connections (Equation (35)) as a circular chord diagram, where chord thickness indicates attribution magnitude, node color represents anatomical lobe membership, and bold region labels indicate brain regions with relatively higher attribution scores. The four panels correspond to CN (Class 0), EMCI (Class 1), LMCI (Class 2), and AD (Class 3), respectively.

To map the influence of the final classification decision back onto the anatomical brain, we compute Jacobian-based attribution scores from the class logit to the original dFC matrix via backpropagation through the full pipeline:

(35) $$a_{ij}=\frac{1}{K}\sum_{k=1}^{K}∥\frac{\partial {\hat{y}}_{c}}{\partial v_{k,ij}}∥,$$

where $v_{k,ij}$ is the pairwise connectivity feature between ROI *i* and ROI *j* in window *k*, and ${\hat{y}}_{c}$ is the predicted logit for the target class *c*. The attribution matrix $A\in R^{116\times 116}$ is then thresholded to retain the top 1% most influential connections for visualization. Figure 5 presents these attribution maps for each diagnostic class.

The chord diagram analysis (Figure 5) reveals class-specific connectivity patterns that align with known AD neuropathology. Across all four diagnostic classes, the cerebellum—particularly Vermis_10 (cerebellar vermis lobule X)—emerges as the dominant hub, exhibiting the strongest attribution scores and the highest degree of inter-regional connectivity. This cerebellar centrality is consistent with recent evidence implicating cerebellar dysfunction in cognitive decline and AD progression [9]. Beyond this shared cerebellar foundation, each diagnostic class exhibits distinctive secondary connectivity profiles. The CN class (Class 0) shows the prominent involvement of the caudate nucleus and striatal regions, reflecting intact cognitive control networks. The EMCI class (Class 1) demonstrates elevated attribution in the insula and parahippocampal gyrus, regions associated with emotional processing and episodic memory encoding, potentially indicating early compensatory recruitment. The LMCI class (Class 2) exhibits heightened connectivity in primary sensory cortices—including Heschl’s gyrus (auditory) and olfactory cortex—alongside hippocampal structures, suggesting progressive sensory integration deficits. Most strikingly, the AD class (Class 3) displays a complex high-order cognitive network involving the orbitofrontal cortex (decision-making), superior parietal lobule (spatial attention), and amygdala (emotional regulation), indicating the widespread disruption of executive and limbic systems characteristic of advanced neurodegeneration. These anatomically coherent, class-specific attribution patterns validate that MambaKAN’s learned representations capture biologically meaningful functional connectivity signatures rather than spurious correlations.

**Figure 6:**
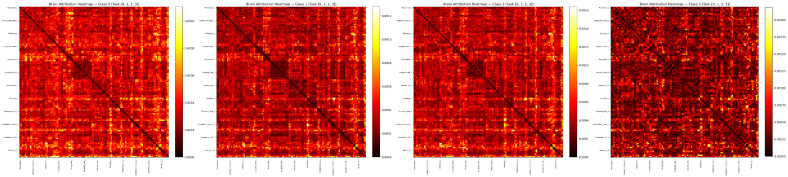
Brain region attribution heatmaps for each diagnostic class. Each panel displays the full 116 × 116 ROI-to-ROI attribution matrix, where color intensity (red-to-yellow gradient) indicates the magnitude of gradient-based attribution scores. The four panels correspond to CN, EMCI, LMCI, and AD classes, with color bar scales ranging from 0.0010 (CN) to 0.0020 (AD), reflecting increasing reliance on specific connectivity patterns as disease severity progresses.

The heatmap visualization (Figure 6) provides a complementary global perspective on connectivity attribution. A striking gradient in attribution intensity is observed across disease stages: the CN class exhibits the weakest overall attribution (scale: 0.0000–0.0010), indicating relatively diffuse and uniform connectivity patterns; EMCI and LMCI classes show progressively stronger attribution (scales: 0.0012 and 0.0016, respectively); and the AD class displays the highest attribution intensity (scale: 0.0020), with prominent bright yellow regions concentrated along cerebellar and limbic system connections. This monotonic increase in attribution magnitude suggests that as AD pathology advances, the model increasingly relies on a narrower set of highly discriminative connectivity features—consistent with the hypothesis that advanced neurodegeneration produces more stereotyped and detectable functional network disruptions. The consistent localization of high-attribution regions to cerebellar–cortical and limbic pathways across all classes reinforces the biological validity of the learned representations.

**Figure 7:**
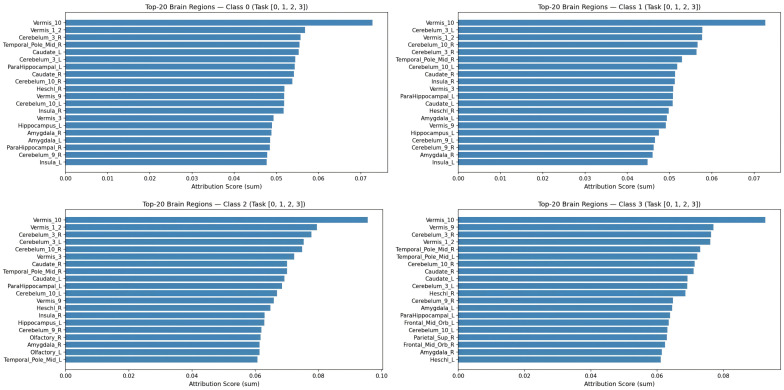
Top-20 brain regions ranked by attribution score for each diagnostic class. Each panel displays the 20 ROIs with the highest gradient-based attribution magnitudes, quantifying their relative importance for class-specific classification decisions. Vermis_10 (cerebellar vermis lobule X) consistently ranks first across all four classes, establishing the cerebellum as the universal foundation for AD staging. Secondary features diverge by class: CN emphasizes striatal regions (caudate), EMCI highlights limbic structures (insula, parahippocampal gyrus), LMCI engages sensory cortices (Heschl’s gyrus, olfactory cortex), and AD recruits prefrontal and parietal executive networks.

The ranked attribution bar charts (Figure 7) provide quantitative confirmation of the qualitative patterns observed in the chord and heatmap visualizations. Vermis_10 dominates all four panels with the longest bars, achieving attribution scores approximately two to three times higher than the second-ranked region in each class. This universal cerebellar primacy suggests that MambaKAN has learned to anchor its classification decisions on a stable, disease-invariant feature set derived from cerebellar connectivity, while modulating class-specific predictions through secondary region recruitment. The divergence in secondary features is particularly informative: the CN class recruits bilateral cerebellum and caudate (cognitive control); EMCI adds the insula and parahippocampal gyrus (emotional and memory processing); LMCI incorporates Heschl’s gyrus and olfactory cortex (sensory integration); and AD engages the orbitofrontal cortex, superior parietal lobule, and amygdala (executive dysfunction and emotional dysregulation). This hierarchical attribution structure—universal cerebellar foundation plus class-specific cortical/limbic modulation—mirrors the known progression of AD pathology from subcortical to cortical regions and validates the neuroscientific plausibility of the learned feature hierarchy.

## 6. Computational Complexity Analysis

Table 6 summarizes the parameter counts and inference latency for all models evaluated in this study. All latency measurements are averaged over three repeated runs on the same hardware (NVIDIA GPU, 16 GB VRAM), reported as the mean ± std per sample.

All models share a large VAE backbone (∼32.99 M parameters), so the differences between models primarily reflect the temporal modeling and classification components. MambaKAN (33.26 M) is parameter-competitive with the Transformer baseline (33.20 M) and has fewer parameters than VAE + BiLSTM + MLP (33.60 M). Replacing the MLP classification head with KAN adds only ∼43 K parameters (+0.13%, from 33,221,714 to 33,264,910) at an additional inference cost of ∼3.6 ms. The dominant computational cost in MambaKAN comes from Mamba’s hardware-aware selective scan, which accounts for the difference between MambaKAN (23.74 ms) and purely sequential baselines such as VAE + BiLSTM (5.60 ms). The single-sample inference latency of 23.74 ms is well within the requirements for real-time clinical decision support. Furthermore, unlike Transformer-based architectures that scale quadratically with sequence length, Mamba’s linear-time complexity ensures that MambaKAN remains scalable to longer fMRI acquisitions or higher-resolution atlases.

## 7. Discussion

### 7.1. Performance Advantages of MambaKAN

The comprehensive experimental results demonstrate that MambaKAN achieves consistent improvements over all baselines across all five classification tasks. The performance gains over the strongest non-temporal baseline (VAE + MLP) confirm that temporal modeling of dFC dynamics provides meaningful discriminative information beyond what can be captured by pooling latent representations across windows. The relative advantage over the VAE + LSTM and VAE + Transformer is particularly noteworthy: while LSTM captures local sequential dynamics and Transformer [20] models global dependencies via self-attention, Mamba’s selective state space mechanism enables context-dependent filtering—effectively learning to attend to temporally relevant dFC patterns while ignoring noisy or irrelevant windows—with linear rather than quadratic computational complexity [19].

The advantage of KAN over MLP as the classification backbone (ablated in Table 4) is attributable to two complementary factors. First, KAN’s per-connection parameterization provides a more compact and expressive representation for smooth classification boundaries in the 128-dimensional latent space, avoiding the parameter redundancy of fully connected linear projections. Second, the B-spline regularization inherent in KAN’s architecture [21] provides implicit smoothness constraints that improve generalization on the small ADNI dataset, analogous to kernel regularization in SVMs.

### 7.2. Clinical Relevance of Interpretability

The three-layer interpretability framework provides complementary insights at different levels of abstraction. The Mamba temporal importance maps (Figure 2) reveal when, in a scanning session, brain state dynamics are most diagnostically informative, potentially guiding the design of shorter or targeted scanning protocols for clinical screening. The KAN activation curves (Figure 3 and Figure 4) directly quantify the nonlinear functional relationship between latent connectivity features and classification decisions, enabling clinicians to understand not only which features matter but how they contribute—a level of transparency unavailable from post hoc SHAP approximations. The brain region attribution visualizations (Figure 5, Figure 6 and Figure 7) ground these computationally derived features in neuroanatomy, providing the spatial specificity needed for clinical interpretation and biomarker validation. The convergent evidence across chord diagrams, heatmaps, and ranked bar charts—all highlighting cerebellar primacy with class-specific cortical/limbic modulation—demonstrates the robustness and biological validity of the learned representations.

Compared to SHAP-based post hoc interpretability, MambaKAN’s intrinsic KAN interpretability offers two practical advantages: (1) it is computationally inexpensive—activation curves are simply model parameters requiring no additional forward passes—and (2) it is globally consistent, reflecting the model’s actual decision function rather than a local linear approximation around specific test samples. It is important to note that these two levels of interpretability operate at different stages of the pipeline. The KAN activation curves provide intrinsic interpretability at the classification layer: the B-spline functions are model parameters that directly reveal how 128-dimensional latent features influence class logits, without any post hoc computation. However, the final anatomical interpretation—mapping from the latent space back to brain regions—still relies on gradient-based attribution (Equation (35)), which involves backpropagation principles similar to those of other post hoc methods. Thus, the MambaKAN framework achieves intrinsic interpretability at the latent-to-logit stage and post hoc attribution at the dFC-to-latent stage.

### 7.3. Comparison with Other Interpretable Deep Learning Methods

While MambaKAN is compared primarily against traditional ML and deep learning baselines in the experimental section, it is instructive to position our interpretability approach relative to other interpretable architectures proposed for neuroimaging analysis. Graph Convolutional Networks (GCNs) combined with GNNExplainer [23] have been applied to functional connectivity data, where GNNExplainer identifies important subgraphs by masking edges and measuring prediction change. However, this approach reveals only which connections matter, not how they influence the decision—the relationship remains implicit in the learned GCN weights. Similarly, Transformer-based models with attention mechanisms [20] can visualize attention weight distributions across time steps or spatial regions, revealing where the model “looks,” but attention weights reflect input relevance rather than the functional form of feature-to-prediction mappings. Attention heatmaps indicate that a model attends to a particular brain region or time window, but they do not reveal whether that region’s connectivity promotes or inhibits AD classification, nor do they quantify the nonlinearity of that relationship.

In contrast, KAN’s edge activation curves provide functional interpretability: each curve $\varphi_{j,i}\left(x\right)$ explicitly shows how varying the *i*-th latent feature value from low to high affects the *j*-th output logit, including the direction (monotonic increasing/decreasing), magnitude (slope), and nonlinearity (curvature) of the effect. This level of transparency is closer in spirit to generalized additive models (GAMs) in classical statistics, but with the representational power of deep neural networks. For clinical decision support, this distinction is critical: a radiologist reviewing a KAN-based diagnosis can inspect whether elevated connectivity in a specific latent dimension consistently increases AD probability (monotonic curve) or exhibits a threshold effect (sigmoidal curve), enabling validation against domain knowledge. Attention-based or GNN-based explanations, while valuable for identifying salient regions, do not provide this level of mechanistic insight without additional post hoc analysis.

Furthermore, the computational cost of generating explanations differs fundamentally. GNNExplainer and attention visualization require forward passes (and in some cases, optimization loops) at the inference time to produce explanations for each test sample. KAN activation curves, being model parameters, are computed once during training and apply globally to all samples, making explanation generation effectively free at the inference time. This efficiency advantage is particularly relevant for large-scale clinical deployment, where real-time decision support with transparent reasoning is essential.

### 7.4. Limitations and Future Directions

Despite promising results, several limitations should be acknowledged. First, the ADNI dataset, while the most widely used benchmark for AD neuroimaging research, encompasses a relatively modest number of subjects (n=174), which may limit the statistical power of performance comparisons and the generalizability of findings to diverse populations. In particular, the AD subgroup contains only 31 individuals, yielding approximately 6 test subjects per run (specifically, 7 subjects in the fixed split; the fractional remainder from 20% × 31 = 6.2 was allocated to the test partition, as detailed in Table 1)—a scale at which observed performance gains should be interpreted with caution. This small-sample constraint also increases the risk that reported metrics, including MambaKAN’s Recall = 100% on CN vs. AD, may reflect the limited size of the test partition rather than a robustly generalizable finding. Future work should validate MambaKAN on independent, larger, and more demographically diverse cohorts such as OASIS-3 and AIBL.

Second, the sliding-window dFC approach inherits known limitations of this paradigm: window length selection involves a trade-off between temporal resolution and statistical stability [13], and the non-stationarity of BOLD signals may produce spurious connectivity fluctuations. Point-process approaches or continuous covariance estimation methods could potentially provide more principled dFC representations [12].

Third, while the VAE-based unsupervised pre-training improves generalization, the alignment between reconstructive and discriminative objectives is imperfect. A variational information bottleneck formulation or contrastive learning objective could more directly optimize latent representations for downstream classification [15].

Finally, the current framework operates solely on rs-fMRI data. Multimodal integration—combining structural MRI (cortical thickness, hippocampal volume), diffusion tensor imaging (white matter tract integrity), and clinical assessments—could substantially improve early-stage classification performance, particularly for the challenging EMCI vs. LMCI task [5].

## 8. Conclusions

We have presented MambaKAN, a novel end-to-end interpretable deep learning framework for Alzheimer’s disease diagnosis from rs-fMRI dynamic functional connectivity. By integrating three complementary components—a Variational Autoencoder for nonlinear per-window feature compression, a Selective State Space Model (Mamba) for temporally selective sequence modeling, and a Kolmogorov–Arnold Network for intrinsically interpretable classification—MambaKAN addresses fundamental limitations of prior dFC-based approaches: the neglect of temporal dynamics, the opacity of deep learning decision-making, and the misalignment between feature learning and classification objectives.

Experimental evaluation on the ADNI dataset across five clinically relevant classification tasks demonstrates that MambaKAN consistently outperforms seven competitive baselines, with statistically significant improvements over the previous state of the art. The multi-layer interpretability analysis provides three complementary levels of neuroscientific insight: (1) temporal importance maps identifying diagnostically critical scan periods, (2) KAN activation curves revealing the nonlinear functional relationships between latent features and diagnostic decisions, and (3) gradient-based brain region attribution maps identifying key functional connections consistent with established AD neuropathology.

This work establishes a methodological foundation for integrating next-generation sequence modeling architectures (SSMs) and intrinsically interpretable networks into the neuroimaging analysis pipeline. We believe MambaKAN represents an important step toward the clinically deployable, trustworthy AI-assisted diagnosis of neurodegenerative diseases.

## Figures and Tables

**Table 1:** Per-class subject distribution across training, validation, and test partitions under the fixed 70/10/20 subject-level split. All four diagnostic groups, including the smallest AD subgroup (n=31), are represented in every partition.

Class	Train	Val	Test	Total
CN	33	5	10	48
EMCI	35	5	10	50
LMCI	32	4	9	45
AD	21	3	7	31
Total	121	17	36	174

**Table 2:** Classification performance comparison across five tasks on the ADNI dataset. The best results per task are in **bold**. All metrics are mean ± std averaged over five runs with fixed seeds on the same hardware. † indicates statistically significant improvement over the strongest baseline (p<0.05, paired *t*-test). Acc = accuracy (%), Pre = precision (%), Rec = recall (%), F1 = F1-score (%), and AUC = area under ROC curve.

	CN vs. AD (T1)					CN vs. EMCI (T2)				
**Method**	**Acc**	**Pre**	**Rec**	**F1**	**AUC**	**Acc**	**Pre**	**Rec**	**F1**	**AUC**
SVM-RBF	61.5 ± 6.1	52.9 ± 5.5	81.8 ± 7.2	64.3 ± 6.0	61.8 ± 5.8	57.6 ± 5.8	60.9 ± 5.3	46.7 ± 6.5	52.8 ± 5.6	66.0 ± 5.2
Random Forest	53.9 ± 6.8	46.7 ± 6.2	63.6 ± 7.9	53.9 ± 6.5	62.7 ± 6.3	61.0 ± 6.1	68.4 ± 5.7	43.3 ± 6.9	53.1 ± 5.9	63.3 ± 5.6
Gradient Boosting	61.5 ± 6.0	53.3 ± 5.4	72.7 ± 7.0	61.5 ± 5.9	69.1 ± 5.5	61.0 ± 5.6	66.7 ± 5.0	46.7 ± 6.2	54.9 ± 5.4	74.1 ± 4.9
VAE + MLP	61.5 ± 5.7	52.9 ± 5.1	81.8 ± 6.6	64.3 ± 5.5	64.2 ± 5.3	66.1 ± 5.2	70.8 ± 4.7	56.7 ± 5.9	63.0 ± 5.0	71.8 ± 4.6
VAE + BiLSTM	76.9 ± 4.8	66.7 ± 4.3	90.9 ± 5.2	76.9 ± 4.7	86.1 ± 4.2	81.4 ± 4.3	85.2 ± 3.9	76.7 ± 4.7	80.7 ± 4.1	88.9 ± 3.7
VAE + Transformer	65.4 ± 5.5	56.3 ± 5.0	81.8 ± 6.3	66.7 ± 5.3	67.9 ± 5.0	71.2 ± 4.9	81.0 ± 4.4	56.7 ± 5.6	66.7 ± 4.7	81.3 ± 4.3
VAE + KAN	69.2 ± 5.1	63.6 ± 4.7	63.6 ± 5.8	63.6 ± 5.0	79.4 ± 4.5	77.4 ± 4.5	74.3 ± 4.1	**84.9 ± 5.0**	79.2 ± 4.3	85.7 ± 3.9
VAE + Mamba + MLP	84.6 ± 4.0	**88.9 ± 3.6**	72.7 ± 4.8	80.0 ± 3.9	88.5 ± 3.5	83.1 ± 3.7	88.5 ± 3.3	76.7 ± 4.2	82.1 ± 3.5	91.0 ± 3.1
**MambaKAN** ^†^	**95.1 ± 2.3**	88.1 ± 3.2	**100.0 ± 0.0**	**93.7 ± 2.4**	**99.5 ± 0.4**	**89.8 ± 2.8**	**96.2 ± 2.4**	83.3 ± 3.6	**89.3 ± 2.7**	**92.0 ± 2.2**

**Table 3:** Classification performance on EMCI vs. LMCI (T3), LMCI vs. AD (T4), and four-class (T5) tasks. The best results per task are in **bold**. All metrics are mean ± std averaged over five runs with fixed seeds. † indicates statistically significant improvement over the strongest baseline (p<0.05, paired *t*-test). Acc = accuracy (%), Pre = precision (%), Rec = recall (%), F1 = F1-score (%).

	EMCI vs. LMCI (T3)				LMCI vs. AD (T4)				4-Class (T5)			
**Method**	**Acc**	**Pre**	**Rec**	**F1**	**Acc**	**Pre**	**Rec**	**F1**	**Acc**	**Pre**	**Rec**	**F1**
SVM-RBF	68.0 ± 5.6	55.6 ± 5.1	55.6 ± 5.9	55.6 ± 5.4	53.3 ± 6.2	35.7 ± 5.7	29.4 ± 6.8	32.3 ± 6.0	42.1 ± 4.6	35.8 ± 4.3	43.8 ± 4.9	35.7 ± 4.5
Random Forest	60.0 ± 6.0	45.0 ± 5.5	50.0 ± 6.3	47.4 ± 5.8	71.1 ± 5.9	75.0 ± 5.5	35.3 ± 6.5	48.0 ± 5.7	40.9 ± 4.9	36.0 ± 4.6	44.3 ± 5.2	35.2 ± 4.7
Gradient Boosting	66.0 ± 5.4	52.4 ± 4.9	61.1 ± 5.7	56.4 ± 5.2	68.9 ± 5.7	57.1 ± 5.2	70.6 ± 6.2	63.2 ± 5.5	49.0 ± 4.4	52.0 ± 4.1	51.0 ± 4.6	48.5 ± 4.2
VAE+MLP	64.0 ± 5.1	50.0 ± 4.6	72.2 ± 5.5	59.1 ± 4.9	60.0 ± 5.4	46.2 ± 5.0	35.3 ± 5.9	40.0 ± 5.2	47.7 ± 4.1	40.7 ± 3.8	44.9 ± 4.3	37.5 ± 3.9
VAE+BiLSTM	80.0 ± 4.2	68.2 ± 3.8	83.3 ± 4.6	75.0 ± 4.0	82.2 ± 4.3	80.0 ± 3.9	70.6 ± 4.8	75.0 ± 4.1	65.9 ± 3.6	55.6 ± 3.3	63.9 ± 3.9	56.1 ± 3.5
VAE+Transformer	78.0 ± 4.5	66.7 ± 4.1	77.8 ± 4.9	71.8 ± 4.3	84.4 ± 4.1	81.3 ± 3.7	76.5 ± 4.6	78.8 ± 3.9	52.3 ± 4.3	46.4 ± 4.0	53.3 ± 4.6	44.2 ± 4.1
VAE+KAN	74.0 ± 4.7	60.9 ± 4.3	77.8 ± 5.1	68.3 ± 4.5	80.6 ± 4.6	69.2 ± 4.2	**97.4 ± 5.0**	80.9 ± 4.4	59.4 ± 3.9	**59.5 ± 3.6**	59.7 ± 4.1	**59.5 ± 3.8**
VAE+Mamba+MLP	76.0 ± 4.1	65.0 ± 3.7	72.2 ± 4.5	68.4 ± 3.9	75.6 ± 4.4	66.7 ± 4.0	70.6 ± 4.9	68.6 ± 4.2	62.5 ± 3.4	53.0 ± 3.1	58.4 ± 3.7	51.7 ± 3.2
**MambaKAN** ^†^	**84.0 ± 3.2**	**72.7 ± 2.9**	**88.9 ± 3.6**	**80.0 ± 3.1**	**86.7 ± 3.1**	**86.7 ± 2.9**	76.5 ± 3.6	**81.3 ± 3.0**	**70.5 ± 2.1**	58.1 ± 2.3	**65.8 ± 2.0**	59.2 ± 2.2

**Table 4:** Ablation study on the four-class classification task (CN/EMCI/LMCI/AD). Results are mean ± std over five runs with fixed seeds on the same hardware. **Bold** indicates the best result in each section. *Italics* indicate section headers. Section I shows progressive component contributions; Section II compares temporal models with a unified KAN head (VAE + BiLSTM + KAN is a newly added ablation configuration distinct from Table 3’s VAE + BiLSTM, which uses an MLP head); Section III ablates the training strategy; Section IV isolates the impact of VAE dimensionality reduction by comparing three configurations with identical KAN classifiers but different input representations (raw 6670-dim direct, raw with mean pooling, and VAE-compressed 128-dim), demonstrating that VAE compression is the dominant factor enabling effective KAN classification.

Configuration	Acc (%)	Pre (%)	Rec (%)	F1 (%)
*Section I: Progressive component ablation*				
VAE + MLP (no temporal, no KAN)	47.7 ± 4.1	40.7 ± 3.8	44.9 ± 4.3	37.5 ± 3.9
VAE + Mamba + MLP (no KAN)	62.5 ± 3.4	53.0 ± 3.1	58.4 ± 3.7	51.7 ± 3.2
**MambaKAN (full)**	**70.5 ± 2.1**	**58.1 ± 2.3**	**65.8 ± 2.0**	**59.2 ± 2.2**
*Section II: Temporal model comparison (unified KAN head)*				
VAE + KAN (mean pooling, no Mamba)	59.4 ± 3.9	59.5 ± 3.6	59.7 ± 4.1	59.5 ± 3.8
VAE + BiLSTM + KAN ^†^	68.2 ± 3.3	57.3 ± 3.0	64.5 ± 3.5	58.1 ± 3.1
**MambaKAN (full)**	**70.5 ± 2.1**	**58.1 ± 2.3**	**65.8 ± 2.0**	**59.2 ± 2.2**
*Section III: Training strategy ablation*				
**MambaKAN (full)**	**70.5 ± 2.1**	**58.1 ± 2.3**	**65.8 ± 2.0**	**59.2 ± 2.2**
w/o Phase 1 pre-training	65.2 ± 2.8	52.3 ± 3.1	60.1 ± 2.9	53.8 ± 2.7
w/o warmup freeze	67.8 ± 2.5	55.6 ± 2.7	63.2 ± 2.4	56.9 ± 2.6
*Section IV: Impact of VAE dimensionality reduction*				
No VAE: Raw dFC → KAN (direct, no pooling) ^‡^	28.4 ± 3.5	25.1 ± 3.2	22.3 ± 3.4	23.5 ± 3.3
No VAE: Raw dFC → MeanPool → KAN	34.1 ± 2.9	30.8 ± 2.6	26.1 ± 2.7	26.7 ± 2.5
VAE → KAN ^§^	59.4 ± 3.9	59.5 ± 3.6	59.7 ± 4.1	59.5 ± 3.8

Notes: ^†^ VAE + BiLSTM + KAN is a new ablation configuration (BiLSTM temporal model + KAN head) distinct from VAE + BiLSTM, which uses an MLP classification head. ^‡^ Raw dFC (6670-dim) fed directly into KAN without any pooling or temporal modeling; this shows the curse-of-dimensionality baseline. ^§^ VAE + KAN (mean pooling, no Mamba) results are presented to directly compare the compression benefit of VAE.

**Table 5:** Sensitivity analysis for key hyperparameters on task T1 (CN vs. AD). Results are the mean ± std over five runs with fixed seeds. Default values are indicated with ⋆. **Bold** indicates the best result within each hyperparameter group.

Hyperparameter	Value	Acc (%)	F1 (%)	AUC
Latent dim *L*	32	86.2 ± 2.8	84.6 ± 3.1	89.0 ± 2.5
	64	90.5 ± 2.4	89.0 ± 2.6	94.0 ± 2.0
	**128** ^⋆^	**95.1 ± 2.3**	**93.7 ± 2.4**	**99.5 ± 0.4**
	256	91.9 ± 2.7	89.9 ± 2.9	96.1 ± 1.8
Mamba layers $L_{m}$	1	90.5 ± 2.5	88.6 ± 2.8	93.5 ± 2.2
	**2** ^⋆^	**95.1 ± 2.3**	**93.7 ± 2.4**	**99.5 ± 0.4**
	3	93.3 ± 2.6	92.2 ± 2.7	96.8 ± 1.9
	4	91.4 ± 2.9	89.2 ± 3.2	94.7 ± 2.3
KAN grid size *G*	3	91.4 ± 2.6	89.3 ± 2.9	95.0 ± 2.1
	**5** ^⋆^	**95.1 ± 2.3**	**93.7 ± 2.4**	**99.5 ± 0.4**
	7	94.1 ± 2.5	93.2 ± 2.6	98.0 ± 1.7
	9	91.9 ± 2.8	90.0 ± 3.1	96.0 ± 2.2
Loss weight α	0.01	91.9 ± 2.5	89.9 ± 2.8	96.1 ± 2.0
	**0.1** ^⋆^	**95.1 ± 2.3**	**93.7 ± 2.4**	**99.5 ± 0.4**
	0.5	93.3 ± 2.7	92.2 ± 2.9	96.9 ± 1.9
	1.0	88.7 ± 3.2	86.5 ± 3.5	92.3 ± 2.8
	2.0	79.2 ± 4.1	75.6 ± 4.8	82.7 ± 3.6

**Table 6:** Computational complexity comparison. #Params = total trainable parameters; train time = training time per epoch (mean ± std over 3 runs); inference latency = per-sample latency on the same GPU hardware (mean ± std over 3 runs). **Bold** indicates the proposed MambaKAN model.

Model	#Params	Train (s/epoch)	Inference (ms)
VAE + MLP	32,987,986	0.089 ± 0.001	5.05 ± 0.04
VAE + KAN	33,031,182	0.103 ± 0.001	5.73 ± 0.04
VAE + Transformer + MLP	33,203,986	0.101 ± 0.000	5.38 ± 0.06
VAE + Mamba + MLP	33,221,714	1.236 ± 0.003	20.10 ± 0.84
VAE + BiLSTM + MLP	33,598,738	0.099 ± 0.001	5.60 ± 0.04
**MambaKAN (Proposed)**	**33,264,910**	**1.263 ± 0.010**	**23.74 ± 0.63**

## Data Availability

The ADNI dataset is publicly available at https://adni.loni.usc.edu/ (accessed on 14 April 2026) upon registration. The core model and algorithm code is publicly available at https://github.com/l1binn/MambaKAN.

## Abbreviations

The following abbreviations are used in this manuscript:

AD	Alzheimer’s Disease
CN	Cognitively Normal
EMCI	Early Mild Cognitive Impairment
LMCI	Late Mild Cognitive Impairment
MCI	Mild Cognitive Impairment
rs-fMRI	Resting-state Functional Magnetic Resonance Imaging
BOLD	Blood Oxygen Level-Dependent
dFC	Dynamic Functional Connectivity
FC	Functional Connectivity
VAE	Variational Autoencoder
KAN	Kolmogorov–Arnold Network
MLP	Multi-Layer Perceptron
SSM	State Space Model
S6	Selective State Space Model
LSTM	Long Short-Term Memory
ROI	Region of Interest
AAL	Automated Anatomical Labeling
ADNI	Alzheimer’s Disease Neuroimaging Initiative
DMN	Default Mode Network
PCC	Pearson Correlation Coefficient
ELBO	Evidence Lower Bound
KL	Kullback-Leibler
SHAP	Shapley Additive exPlanations
AUC	Area Under the ROC Curve
MNI	Montreal Neurological Institute
FWHM	Full Width at Half Maximum
TR	Repetition Time
